# A Novel Nonenzymatic Hydrogen Peroxide Sensor Based on a Polypyrrole Nanowire-Copper Nanocomposite Modified Gold Electrode

**DOI:** 10.3390/s8085141

**Published:** 2008-08-28

**Authors:** Tingting Zhang, Ruo Yuan, Yaqin. Chai, Wenjuan Li, Shujuan Ling

**Affiliations:** Chongqing Key Laboratory of Analytical Chemistry, College of Chemistry and Chemical Engineering, Southwest University, key Laboratory on Luminescence and Real-Time Analysis Southwest University, Ministry of Education, Chongqing 400715, P.R. China; E-Mails: ztt8208@swu.edu.cn (T.T.Z.); yqchai@swu.edu.cn (Y.Q.C); ruyue@swu.edu.cn (W.J.L); lsj0832@swu.edu.cn (S.J.L)

**Keywords:** Polypyrrole nanowires, Copper nanoparticles, Nonenzymatic sensor, Hydrogen peroxide

## Abstract

A novel nonenzymatic hydrogen peroxide (H_2_O_2_) sensor has been fabricated by dispersing copper nanoparticles onto polypyrrole (PPy) nanowires by cyclic voltammetry (CV) to form PPy-copper nanocomposites on gold electrodes. Scanning electron microscopy (SEM) was used to characterize the morphologies of the PPy nanowires and the PPy-copper nanocomposite. The reactivity of the PPy-copper nanocomposite towards H_2_O_2_ was characterized by cyclic voltammetry and chronoamperometry. Effects of applied potential, the concentrations of detection solution upon the response currents of the sensor were investigated for an optimum analytical performance. It was proved that the PPy-copper nanocomposite showed excellent catalytic activity for the reduction of hydrogen peroxide (H_2_O_2_). The sensor showed a linear response to hydrogen peroxide in the concentration range between 7.0×10^-6^ and 4.3×10^-3^ mol L^-1^ with a high sensitivity, and a detection limit of 2.3×10^-6^ mol L^-1^. Experiment results also showed that the sensor had good stability.

## 1. Introduction

There is a need for economical, simple and reliable methods to detect hydrogen peroxide (H_2_O_2_), because of its use in many research fields such as the food industry, biotechnology, the clinic, the pharmaceutical industry and environmental protection [1, 3]. Many analytical methods have been reported for the determination of H_2_O_2_ [4], including spectrophotometry [5], chemiluminescence [6], and electrochemistry [7-9]. Among these methods, electrochemistry has become a subject of considerable interest because of its low detection limit, high selectivity and high sensitivity. Many of these biosensors were based on immobilization of a protein, such as horseradish peroxidase (HRP) [10], hemoglobin (Hb) [11, 12] and heme [13, 14] for detecting H_2_O_2_, but the ready denaturation of immobilized enzyme/protein on the surface of the electrode is a common problem which leads to such modified electrodes suffering from a poor enzyme/protein activity and low reproducibility and stability [15]. Considering these facts, there has been more and more interest in nonenzymatic sensors and the fabrication of nonenzymatic sensors, including electrodes modified with bismuth [16], carbon nanotubes [17] and conducting polymers [18] has been reported.

Pyrrole, as a key member within the organic conducting polymers family, has higher conductivity than many other conducting polymers such as polyaniline, as well as good environmental stability [19]. For these reasons it has attracted considerable attention and many articles have reported its applications in biosensors [20, 21]. PPy film could be further improved by embedding metal particles into the polymer matrix to form a metal–polymer composite [22, 23]. This polymer-metal nanocomposite can provide a highly porous structure with a large effective surface area, good electronic conductivity and high catalytic activity [24]. Some metal–polymer nanocomposites have already been reported in the literature, such as PPy/Au [25], PPy/Pt [26], PPy/Ag [27], PPy/Ti [28] and PPy/Pd nanocomposites [29].

PPy nanowires the subject of a great deal of interest because they offer more advantages than traditionally synthesized PPy films, such as their higher electronic conductivity, charge transport properties, well-ordered polymer chain structures with high surface-to-volume ratio and small cross dimensions [30]. Tian *et al*. have prepared PPy nanowires on electrode surfaces under stationary potentials by a template-free method to produce an enzymatic biosensor [31]. Li *et al*. have reported Pt nanoclusters embedded in PPy nanowires to fabricate glucose biosensors [24].

It is well known that the copper-based chemically modified electrodes have been used in fabricating sensors. A hydrogen peroxide biosensor is fabricated by using a DNA–Cu (II) complex as electrocatalyst [32]. Copper-dispersed polyaniline modified electrode is capable of oxidizing glucose in an alkaline hydroxide solution [33]. Glucose sensor is fabricated with a composite of copper nanocluster/multiwall carbon nanotube [34]. Dimethylglyoxime functionalized copper nanoparticles (DMG-CuNPs) were synthesized by a simple microwave irradiation method [35]. The characterization of polypyrrole film modified with copper nanoparticles has been analyzed by Cioffi [36], but there are no reports on the application of the nanocomposite of copper nanoparticles dispersed onto PPy nanowires to construct a hydrogen peroxide sensor.

In previous work, we have developed some biosensors based on multiwall carbon nanotube/gold nanoparticles and silver nanoparticles to immobilize Hb and HRP for detection hydrogen peroxide [37, 38]. This paper describes a simple and effective method to fabricate a nonenzymatic hydrogen peroxide sensor by catalytic reduction with electropolyrized copper nanoparticles on the electrode modified with PPy nanowires. Though lacking an enzyme film, the sensor exhibited excellent performance features, such as low detection limits, wide linear range, quick current response, high sensitivity and good stability. This may be due to the PPy-copper nanocomposite providing a large surface area, good electronic conductivity and high catalytic activity.

## 2. Results and Discussion

### 2.1 Characterization of Electrode Surface

**Figure 1.**
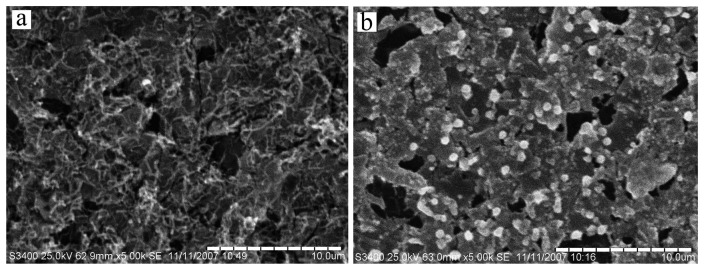
SEM images of PPy nanowires (a) and PPy–copper nanocomposite surface (b) on gold electrode.

The morphologies of PPy nanowires and PPy-copper nanocomposite were investigated by scanning electron microscopy (SEM). Figure 1(a) show the fibriform morphology PPy nanowires.The PPy nanowires film possessed gaps and pores, so it was easy for copper nanoparticles to disperse on the special structure film. Figure 1(b) shows the morphologies of the PPy-copper nanocomposite used in the experiment. When copper nanoparticles were polymerized on the PPy nanowires film, the PPy nanowires in Figure 1(b) was not clear enough due to that the nanowires were covered with copper nanoparticles.

### 2.2 Electrochemical Characterization of the Modified Electrode

**Figure 2.**
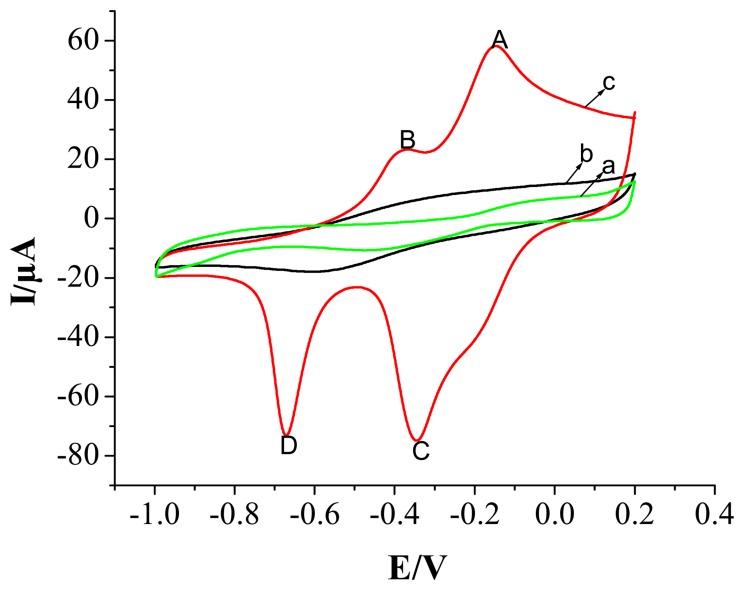
Cyclic voltammograms of an Au electrode in NaOH (0.1 mol L^-1^). (a) bare Au electrode, (b) PPy nanowires modified electrode and (c) PPy-copper nanocomposite modified electrode by sweeping at a scan rate of 100 mV s^-1^ in a 0.1 mol L^-1^ NaOH solution.

Cyclic voltammetry (CV) was useful in providing reliable chemical information of the electrode in alkaline solution. Figure 2 shows cyclic voltammograms of different modified electrodes in NaOH (0.1 mol L^-1^) solution. Figure 2a shows the graph of a clean Au electrode surface. When the electrode was modified with PPy nanowires, no obvious peaks appeared (Figure 2b). After the electrode was modified with copper nanoparticles, two pairs of oxidation and reduction peaks were observed and the current was increased (Figure 2c). The anodic peaks labeled A at -0.152 V represent the transition of Cu (I) to Cu (II), The anodic peaks labeled B at the -0.378 V the formation of Cu (II) species due to oxidation of metallic Cu to Cu (II) and Cu (I) to Cu (II). The cathodic peaks labeled C at the -0.345 V and D at the -0.674 V correspond to the transition of Cu (II) to Cu (I), Cu (I) to Cu (0), respectively. These CV characteristies of the modified electrode were similar to that reported in the literatures [34, 35]. But, the peak potentials observed at the composite are slightly shifted in the positive direction.

### 2.3 Influence of Potential on Sensor Response

**Figure 3.**
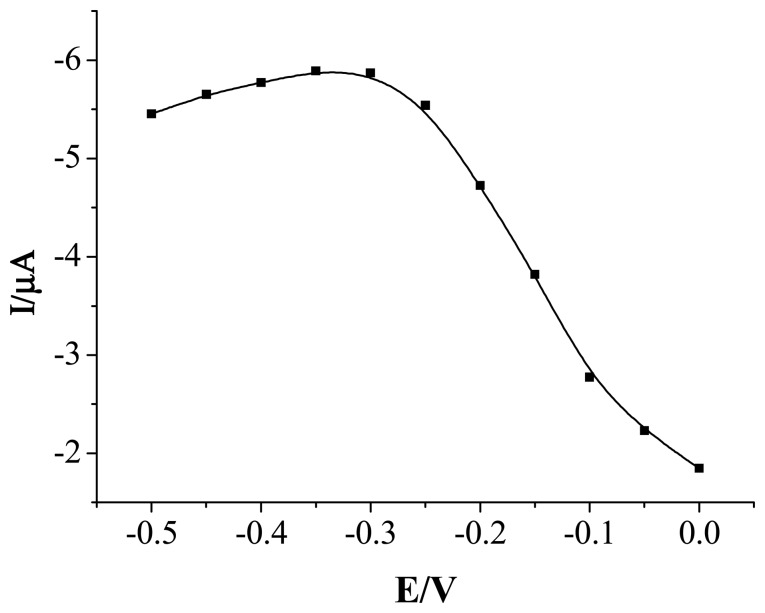
Effect of the work potential to PPy-Cu nanocomposite modified Au electrode in the presence of 1.4×10^-5^ mol L^-1^ H_2_O_2_. Applied potential: -0.3 V. Solution: 0.1 mol L^-1^ NaOH.

In order to improve the performance of the sensor, factors which may influence the response of the sensor were studied. Figure 3 shows the dependence of the chronoamperometric current response to constant concentration 1.4×10^-5^ mol L^-1^ H_2_O_2_ on the applied potential in the range from 0 V to -0.5 V. As can be seen, the response current increased from 0 to -0.35 V. When the then potential is more negative than -0.35 V, the response current decreases slightly. To decrease the contribution from the most common interferents, a potential of -0.3 V was choosen as work potential in all the subsequent amperometric detection.

### 2.4 Optimization of the Concentration of NaOH for the Sensor

To enhance the electrocatalytic activity of PPy-Cu nanocomposite for hydrogen peroxide, an alkaline medium is required. When the concentrations of the NaOH were changed from 40 mmol L^-1^ to 100 mmol L^-1^, the response current was increased. But, when the concentrations of NaOH were above 100 mmol L^-1^, the response current was not improved with high background noise. In this experiment, 100 mmol L^-1^ NaOH was choosen as the detection solution.

### 2.5 The Sensor Response to Hydrogen Peroxide

**Figure 4.**
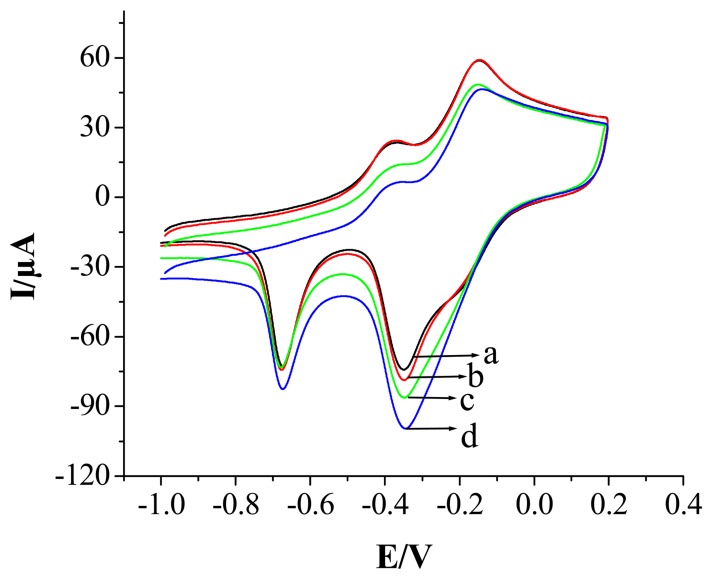
Cyclic voltammograms of the Ppy-copper nanocomposite modified Au electrode in the presence of different H_2_O_2_ concentration in the solution of 0.1 mol L^-1^ NaOH at 100 mV s^-1^, (a) 0 mol L^-1^, (b) 3.5×10^-5^ mol L^-1^, (c) 4.9×10^-4^ mol L^-1^ and (d)1.19×10^-3^ mol L^-1^.

Figure 4 shows cyclic voltammograms of the PPy-copper nanocomposite modified Au electrode without and with different H_2_O_2_ concentration in the solution of 0.1 mol L^-1^ NaOH at 100 mV s^-1^. In the absence of H_2_O_2_ a typical PPy-Cu nanocomposite oxidation and reduction peak was observed (curve a). When 3.5×10^-5^ mol L^-1^ H_2_O_2_ was added to NaOH (0.1 mmol L^-1^) solution, an obvious increase of the cathodic peak current was observed (curve b), indicating that the PPy-Cu nanocomposite showed good catalysis towards H_2_O_2_. With the addition of H_2_O_2_, the cathodic peak current increased significantly and the anodic peak current decreased obviously (curve b-d). It was observed that reduction peak current increased with the increasing concentration of H_2_O_2_. However, no catalytic current corresponding to the reduction of H_2_O_2_ can be observed at PPy nanowires modified electrode under the same condition, so it can be concluded that copper ion in the PPy-Cu nanocomposite is responsible for the reduction of H_2_O_2_. In a possible catalytic mechanism for the reduction of H_2_O_2_ Cu (II) was first reduced electrochemically to Cu (I), which reacted chemically with H_2_O_2_ and resulted in the conversion of H_2_O_2_ into OH^-^ and regeneration of the catalyst, as shown in the following equations:

(1) $$\text{PPy}/\text{Cu}\;(\text{II})+{\text{e}}^{‐}\to \text{PPy}/\text{Cu}\;(\text{I})$$

(2) $$\text{PPy}/\text{Cu}\;(\text{I})+1/2\;{\text{H}}_{2}{\text{O}}_{2}\to \text{PPy}/\text{Cu}\;(\text{II})+{\text{OH}}^{‐}$$

**Figure 5.**
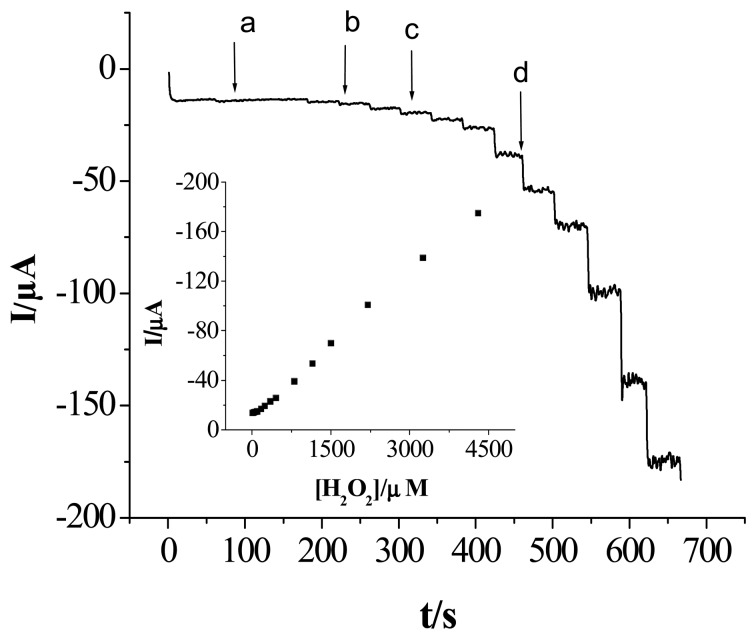
Shows the typical current-time curves of the sensor under the applied potential of -0.3 V with successive injection of H_2_O_2_ in a stirred 0.1 mol L^-1^ NaOH solution. The injection of H_2_O_2_ concentration: (a) 7.0 ×10^-6^ mol L^-1^, (b) 2.8 ×10^-5^ mol L^-1^, (c) 9.8 ×10^-5^ mol L^-1^ and (d) 4.48 ×10^-4^ mol L^-1^. Inset shows linear calibration curves.

Figure 5 shows the amperometric response of the sensor under the optimized experiment conditions with successive addition of different concentrations of hydrogen peroxide in a stirred 0.1 mol L^-1^ NaOH solution.

At an applied potential of -0.3 V, as the concentration of H_2_O_2_ increased the response current increased immediately and finally reached 95% of the steady-state value; a fast response time of 5 s was estimated. Such a fast response time may be attributed to fast diffusion of H_2_O_2_ within the PPy-copper nanocomposite and excellent electron transfer behavior of copper within the PPy film on the gold electrode surface. We used the steady-state current to plot with the concentration of H_2_O_2_, as shown in the inset of Figure 5. The sensor displayed a linear range (7.0×10^-6^ to 4.3×10^-3^ mol L^-1^ H_2_O_2_) with a correlation coefficient of 0.9981, a detection limit of 2.3×10- mol L^-1^ at signal-to-noise ration of 3. The good performance of the sensor may be attributed to the good electronic conductivity and high catalytic activity of the PPy-copper nanocomposite.

### 2.6 Stability of the Hydrogen Peroxide Sensor

In order to study the stability of the sensor, amperometric measurements were performed in the presence of 1.0×10^-4^ mol L^-1^ H_2_O_2_ periodically. When not in use, the electrode was stored at 4 □ in a refrigerator. After storage for 1 week, the response of the sensor was maintained about 93 % of the initial values. The sensor still retained 85 % of its original values after two weeks. The storage stability may be attributed to the stable film of the PPy-copper nanocomposite.

### 2.7 Selectivity of the Hydrogen Peroxide Sensor

Selectivity is another important factor which affects the performance of a sensor. In this experiment, five interfering substances (glucose, glycine, ethanol, acetic acid, and l-cysteine) were used to evaluate the selectivity of the sensor. The interference experiments were performed under optimum condition by comparing the current response to 0.2 mmol L^-1^ H_2_O_2_ in the presence of 0.4 mM of each interfering substance with that to 0.2 m mol L^-1^ H_2_O_2_ alone. The results of the interference study are listed in Table 1, the tested substances did not interfere significantly with the resulting sensor.

### 2.8 Recovery Experiment

The application of the sensor was evaluated through detecting recovery. Table 2. shows the recovery of three samples of different H_2_O_2_ concentrations which were derived by standard addition method, the recovery rate between 96.8 % and 105 %.

## 3. Experimental Section

### 3.1 Reagents

Pyrrole was obtained from Shanghai Chemical Reagent Factory and purified twice by distillation under high purity nitrogen and then kept in a refrigerator before use. CuSO_4_·5H_2_O was purchased from Chongqing Chemical Reagent Factory. Hydrogen peroxide (30% w/v solution) was obtained from Chemical Reagent Company, Chongqing, China. The concentration of the more diluted hydrogen peroxide solutions prepared from 30% hydrogen peroxide was determined by titration with potassium permanganate. The solutions of various concentrations of NaOH were prepared for the study. All other Chemicals were of analytical-reagent grade and used without further purification. Doubly distilled water and high purity N_2_ were used.

### 3.2 Apparatus and Chemicals

Electrochemical measurements were carried out on CHI 660A electrochemical workstation (CHI instruments, Chenhua Corp, Shanghai, China). The scanning electron micrographs were taken with a scanning electron microscope (SEM, S-3400, Japan) at an acceleration voltage of 25 kV. A conventional three electrode system was employed with a modified Au electrode as a working electrode, a saturated calomel electrode (SCE) as a reference electrode, and a platinum wire as an auxiliary electrode. All the potentials given in this paper were referred to the SCE. The experimental solutions were deaerated by highly pure nitrogen for 10 min. All the electrochemical experiments were carried out at room temperament.

### 3.3 The modification of the Electrode

**Scheme 1.**
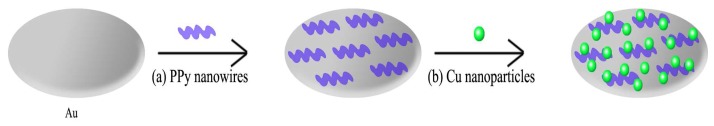
The stepwise fabrication processes of the modified electrode.

The bare Au electrode was polished successively with 0.3 μm and 0.05 μm alumina before modification, and sonicated in double distilled, acetone and double distilled water for 5 min, in order to remove any adsorbed substances on the electrode surface. The PPy-copper nanocomposite was formed in a two-step procedure. In the first stage, The PPy nanowire was electrochemically deposited at a constant potential of 0.80 V for 120 s in an aqueous solution of 0.1 mol L^-1^ LiClO_4_ and 0.1 mol L^-1^ carbonate containing 0.15 mol L^-1^ pyrrole. The electrode was then put into 0.1 mol L^-1^ HClO_4_ solution for 12 h to remove any carbonate ions to obtain the PPy nanowires modified electrode [31]. The second stage, the PPy nanowires modified electrode was immersed in the mixed solution of 0.1 mol L^-1^ Na_2_SO_4_ and 20 mmol L^-1^ CuSO_4_ which was deoxygenated by high purity nitrogen for 10 min and conditioned by cyclic sweeping between -0.40 V to 0.80 V at 100 mV s^-1^ for 30 cycles to obtain the PPy nanowires-copper nanoparticles modified electrode [33, 34]. The fabricated procedure of the sensor was shown in Scheme 1.

## 4. Conclusions

In this paper, a novel nonenzyme hydrogen peroxide biosensor was fabricated by using PPy–copper nanocomposite as a catalyst for the reduction of hydrogen peroxide. It proved that the PPy-copper nanocomposite showed excellent catalysis towards hydrogen peroxide in alkaline media and the sensor showed excellent performances, such as low detection limit, wide linear range, quick current response, high current and good stability. This nonenzyme system also overcame disadvantages of enzyme based biosensor.

## Figures and Tables

**Table 1.** Possible interferences tested by the sensor.

**Interfering reagent**	**Current ratio**^a^^,^^b^

glucose	1.02
glycine	1.01
ethanol	1.01
acetic acid	0.99
l-cysteine	0.96

a Ratio of currents for mixtures containing 0.4 mmol L^-1^ interfering substance and 0.2 mmol L^-1^ H_2_O_2_ to that for 0.2 mmol L^-1^ H_2_O_2_ alone.

b Average values from three successive determinations.

**Table 2.** The recovery of different H_2_O_2_ concentrations in samples tested by the sensor.

**Sample H_2_O_2_** **(mmol L^-1^)**	**Added H_2_O_2_** **(mmol L^-1^)**	**Detected H_2_O_2_** **(mmol L^-1^)**	**Recovery** **(%)**

0.08	0.024	0.106	101.9
0.4	0.35	0.726	96.8
1.2	0.4	1.68	105

## References

[1] Bartlett P.N., Birkin P.R., Wang J.H., Palmisano F., Benedetto G.D. (1998). An enzyme switch employing direct electrochemical communication between horseradish peroxidase and a poly (aniline) film. Anal. Chem..

[2] Wang J., Lin Y.H., Chen L. (1993). Organic-phase biosensor for monitoring phenol and hydrogen peroxide in pharmaceutical antibacterial products. Analyst.

[3] Sellers R.M. (1980). Spectropohotometric determination of hydrogen peroxide using potassium titanium (IV) oxalate. Analyst.

[4] Nakabayashi Y., Yoshikawa H. (2000). Amperometric Biosensors for Sensing of Hydrogen Peroxide Based on Electron Transfer between Horseradish Peroxidase and Ferrocene as a Mediator. Anal. Sci..

[5] Matsubara C., Kawamoto N., Takamura K. (1992). Oxo[5,10,15,20-tetra (4-pyridyl)porphyrinato] titanium (IV): an ultra-high sensitivity spectrophotometric reagent for hydrogen peroxide. Analyst.

[6] Hanaoka S., Lin J.M., Yamada M. (2001). Chemiluminescent flow sensor for H_2_O_2_ based on the decomposition of H_2_O_2_ catalyzed by cobalt (II)-ethanolamine complex immobilized on resin. Anal. Chim. Acta.

[7] Li J., Tan S.N., Ge H.L. (1996). Silica sol–gel immobilized amperometric biosensor for hydrogen peroxide. Anal. Chim. Acta.

[8] Garguilo M.G., Huynh N., Proctor A., Michael A.C. (1993). Amperometric sensors for peroxide, choline, and acetylcholine based on electron transfer between horseradish peroxidase and a redox polymer. Anal. Chem..

[9] Xiao Y., Ju H.X., Chen H.Y. (1999). A reagentless hydrogen peroxide sensor based on incorporation of horseradish peroxidase in poly (thionine) film on a monolayer modified electrode. Anal. Chim. Acta.

[10] Xiao Y., Ju H.X., Chen H.Y. (2000). Direct electrochemistry of horseradish peroxidase immobilized on a colloid/cysteamine-modified gold electrode. Anal. Biochem..

[11] Zhang J.D., Oyama M. (2004). A hydrogen peroxide sensor based on the peroxidase activity of hemoglobin immobilized on gold nanoparticles-modified ITO electrode. Electrochim. Acta.

[12] Wang Q.L., Lu G.X., Yang B.J. (2004). Hydrogen peroxide biosensor based on direct electrochemistry of hemoglobin immobilized on carbon paste electrode by a silica sol–gel film. Sens. Actuat. B.

[13] Liu X.J., Chen T., Liu L.F., Li G.X. (2006). Electrochemical characteristics of heme proteins in hydroxyethylcellulose film. Sens. Actuators B.

[14] Feng J.J., Zhao G., Xu J.J., Chen H.Y. (2005). Direct electrochemistry and electrocatalysis of heme proteins immobilized on gold nanoparticles stabilized by chitosan. Anal. Biochem..

[15] Yang Y., Mu S. (1997). Bioelectrochemical responses of the polyaniline horseradish peroxidase electrodes. J. Electroanal. Chem.

[16] Wittstock G., Strubing A., Szargan R., Werner G. (1998). Glucose oxidation at bismuth-modified platinum electrodes. J. Electroanal. Chem..

[17] Fumiyo K., Satoshi K. (2006). Electrocatalytic activity of bamboo-Structured carbon nanotubes paste electrode toward hydrogen peroxide. Anal. Lett.

[18] Lu Q., Zhou T., Hu S.S. (2007). Direct electrochemistry of hemoglobin in PHEA and its catalysis to H_2_O_2_. Biosens. Bioelectron.

[19] Ashwell G.J. (1992). Molecular Electronics.

[20] Shin M.C., Kim H.S. (1995). Effects of enzyme concentration and film thickness on the analytical performance of a polypyrrole/glucose oxidase biosensor. Anal. Lett.

[21] Fiorito P. A., Brett C.M.A., Torresi S.C. (2006). Polypyrrole/copper hexacyanoferrate hybrid as redox mediator for glucose biosensors. Talanta.

[22] Strike D.J., Rooij N. F. D., Koudelka-Hep M., Ulmann M., Augustynski J. (1992). Electrocatalytic oxidation of methanol on platinum microparticles in polypyrrole. J. Appl. Electrochem..

[23] Rau J.R., Chen S.C., Sun H.W. (1994). Characterization of a polypyrrole microsensor for nitrate and nitrite ions. Electrochim. Acta.

[24] Li J., Lin X.Q. (2007). Glocose biosensor based on immobilization of glucose oxidase in poly(o-aminophenol) film on polypyrrole-Pt nanocomposite modified glassy carbon electrode. Biosens. Bioelectron.

[25] Chen W., Li C.M. (2007). Electrosynthesis and characterization of polypyrrole/Au nanocomposite. Electrochim. Acta.

[26] Bose C.S.C., Rajeshwar K. (1992). Efficient electrocatalyst assemblies for proton and oxygen reduction: the electrosynthesis and characterization of polypyrrole films containing nanodispersed platinum particle. J. Electroanal. Chem..

[27] Liu Y.C., Lee H.T., Yang S.J. (2006). Strategy for the syntheses of isolated fine silver nanoparticles and polypyrrole/silver nanocomposites on gold substrates. Electrochim. Acta.

[28] Roux S., Soler-Illia G.J., Champagne S., Audebert P., Sanchez C. (2003). Titania /Polypyrrole Hybrid Nanocomposites Built from In-Situ Generated Organically Functionalized Nanoanatase Building Blocks. Adv. Mater..

[29] Cioffi N., Torsi L., Losito I., Franco C. D., Bari I.D., Chiavarone L., Scamarcio G., Tsakova V., Sabbatini L., Zambonin P.G. (2001). Electrosynthesis and analytical characterization of polypyrrole thin films modified with copper nanoparticles. J. Mater. Chem..

[30] Li J., Lin X.Q. (2007). Electrocatalytic reduction of nitrite at polypyrrole nanowire– platinum nanocluster modified glassy carbon electrode. Microchem. J..

[31] Tian Y., Wang J.X., Wang Z., Wang S.C. (2005). Solid-phase extraction and amperometric determination of nitrite with polypyrrole nanowire modified electrodes. Sens. Actuat. B.

[32] Gu T.T., Hasebe Y. (2006). DNA–Cu (II) poly(amine) complex membrane as novel catalytic layer for highly sensitive amperometric determination of hydrogen peroxide. Biosens. Bioelectron..

[33] Farrell S. T., Breslin C. B. (2004). Oxidation and photo-induced oxidation of glucose at a polyaniline film modified by copper particles. Electrochim. Acta.

[34] Kang X.H., Mai Z.B., Zou X.Y., Cai P.X., Mo J.Y. (2007). A sensitive nonenzymatic glucose sensor in alkaline media with a copper nanocluster/multiwall carbon nanotube-modified glassy carbon electrode. Anal. Bioanalytical. Chem..

[35] Xu Q., Zhao Y., Xu J.Z. (2006). Preparation of functionalized copper nanoparticles and fabrication of a glucose sensor. Sens. Actuat. B.

[36] Cioffi N., Torsi L., Sabbatini L., Zambonin P.G., Bleve-Zacheo T. (2000). Electrosynthesis and characterisation of nanostructured palladium–polypyrrole composites. J. Electroanal.Chem..

[37] Chen S.H., Yuan R., Chai Y.Q., Zhang L.Y., Wang N., Li X.L. (2007). Amperometric third-generation hydrogen peroxide biosensor based on the immobilization of hemoglobin on multiwall carbon nanotubes and gold colloidal nanoparticles. Biosens. Bioelectron..

[38] Wang F.C., Yuan R., Chai Y.Q., Tang D.P. (2007). Probing traces of hydrogen peroxide by use of a biosensor based on mediator-free DNA and horseradish peroxidase immobilized on silver nanoparticles. Anal. Bioanal. Chem..

